# Protein stabilization utilizing a redefined codon

**DOI:** 10.1038/srep09762

**Published:** 2015-05-18

**Authors:** Kazumasa Ohtake, Atsushi Yamaguchi, Takahito Mukai, Hiroki Kashimura, Nobutaka Hirano, Mitsuru Haruki, Sosuke Kohashi, Kenji Yamagishi, Kazutaka Murayama, Yuri Tomabechi, Takashi Itagaki, Ryogo Akasaka, Masahito Kawazoe, Chie Takemoto, Mikako Shirouzu, Shigeyuki Yokoyama, Kensaku Sakamoto

**Affiliations:** 1 Division of Structural and Synthetic Biology, RIKEN Center for Life Science Technologies, 1-7-22 Suehiro-cho, Tsurumi, Yokohama 230-0045, Japan; 2 RIKEN Systems and Structural Biology Center, 1-7-22 Suehiro-cho, Tsurumi, Yokohama 230-0045, Japan; 3 Department of Chemical Biology and Applied Chemistry, College of Engineering, Nihon University, Koriyama, Fukushima 963-8642, Japan; 4 Biomedical Engineering Research Organization, Tohoku University, Aoba-ku, Sendai 980-8575, Japan; 5 RIKEN Structural Biology Laboratory, 1-7-22 Suehiro-cho, Tsurumi, Yokohama 230-0045, Japan

## Abstract

Recent advances have fundamentally changed the ways in which synthetic amino acids are incorporated into proteins, enabling their efficient and multiple-site incorporation, in addition to the 20 canonical amino acids. This development provides opportunities for fresh approaches toward addressing fundamental problems in bioengineering. In the present study, we showed that the structural stability of proteins can be enhanced by integrating bulky halogenated amino acids at multiple selected sites. Glutathione *S*-transferase was thus stabilized significantly (by 5.2 and 5.6 kcal/mol) with 3-chloro- and 3-bromo-l-tyrosines, respectively, incorporated at seven selected sites. X-ray crystallographic analyses revealed that the bulky halogen moieties filled internal spaces within the molecules, and formed non-canonical stabilizing interactions with the neighboring residues. This new mechanism for protein stabilization is quite simple and applicable to a wide range of proteins, as demonstrated by the rapid stabilization of the industrially relevant azoreductase.

Protein engineering has been facilitated by recombinant technology, which exploits the genetic code of the host organisms to synthesize proteins and generate variations in the amino-acid sequences. However, this dependence on the natural code limits the biotechnology to the 20 canonical amino acids. Including non-natural amino acids in the code expands the entire range of possible permutations in the amino-acid sequences, with the installed novel structural and chemical diversity. There are mainly two cell-based methods for synthesizing proteins with an extra repertoire of amino acids^1^,^2^,^3^. The “residue-specific” method substitutes synthetic amino acids for one of the canonical amino acids in proteins, and the resulting total replacement of the canonical molecule can affect protein stability and other properties. In constrast, the “site-specific” method uses a codon that is not naturally assigned to a canonical amino acid, such as a stop codon, to incorporate synthetic amino acids at any desired sites. However, the low incorporation efficiency, partly due to the existence of the endogenous molecules recognizing the codon assigned to the synthetic molecules, hampers their incorporation at multiple specific sites.

Release factor 1 (RF-1) is the bacterial component recognizing the UAG triplet as a translation stop^4^. RF-1 has been eliminated from *Escherichia coli*, and the *in vivo* meaning of UAG has thus been redefined from a stop codon to a sense codon specific for synthetic amino acids^5^,^6^,^7^,^8^. The codon redefinition has fundamentally changed the manners in which non-natural amino acids are incorporated into proteins, enabling their incorporation at multiple specific sites, in addition to the 20 canonical molecules. Bioengineering based on this development has started to emerge, but presently little is known about what types of effects the novel components exert on protein structures and properties. In the present study, we applied the artificial codes to enhance the versatility of protein engineering. The structural stability of proteins represents a major challenge to rational approaches, because the stability is based on multiple interactions between the amino-acid residues, and thus desirable amino-acid changes cannot be easily designed^9^. We showed that artificial codes provide a simple solution to this problem. The underlying mechanism, inaccessible within the limits of the conventional methods, is now applicable to a wide range of proteins.

## Results and Discussion

### Enhanced structural stabilities of halogenated GST variants

We previously synthesized an iodinated variant (7iGST) of eukaryotic glutathione *S*-transferase (GST), a homodimeric detoxification enzyme, in which seven specific residues of the 15 tyrosines per monomer were replaced by 3-iodo-l-tyrosines^6^. The seven sites, specified with seven UAG codons in the gene, were distributed globally in the enzyme, including four on the surface (positions −1, 22, 141, and 163), two buried (positions 57 and 155), and one at the dimer interface (position 73) (Supplementary Table 1). These sites included all four of the surface tyrosine positions, with the other three sites chosen from the remaining tyrosine positions, except for the catalytically important position 6. *E. coli* RFzero-iy cells were employed to synthesize 7iGST; this strain lacks RF-1, and instead expresses a UAG-reading tRNA derived from *Methanocaldococus jannaschii* tRNA^Tyr^, together with the tyrosyl-tRNA synthetase from the same archaeon engineered to be specific for 3-halogenated tyrosine derivatives^10^. In the present study, two additional variants (7bGST and 7cGST), which contained 3-bromo- and 3-chlorotyrosines, respectively, in place of 3-iodotyrosine, were also synthesized in RFzero-iy, with these tyrosine derivatives being supplemented in the growth media. The yields of 7iGST, 7bGST, and 7cGST were almost the same as that of the wild-type GST (wtGST), and mass spectrometric analyses revealed that these products were homogenous, with each containing the expected number of corresponding halogen atoms (Supplementary Fig. 1).

**Figure 1:**
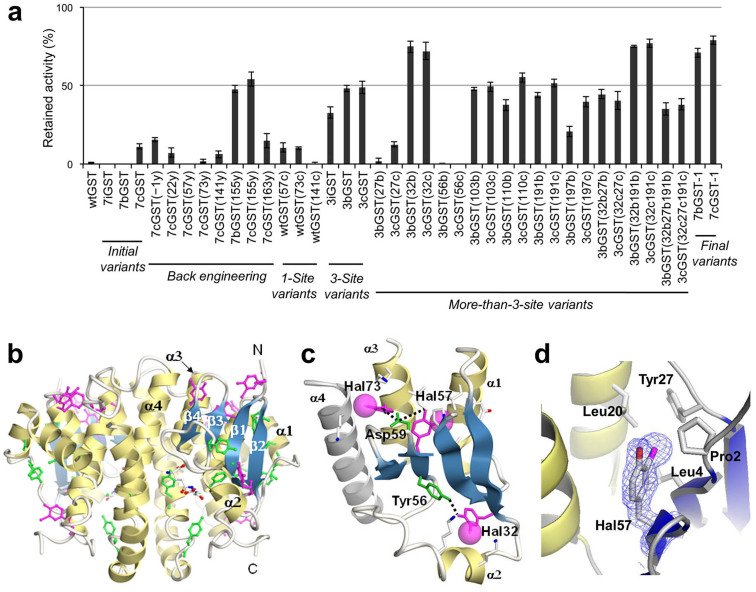
Protein stabilization by multiple selective-site integrations of halogenated tyrosines. (a) The activities retained after heating are indicated for wtGST and the variants. The additional modifications are shown in parentheses, in terms of the engineered site and the final amino acid [tyrosine (y), bromotyrosine (b), and chlorotyrosine (c)]. Error bars indicate standard deviations from three independent measurements for each variant. (b) The crystal structure of the 7bGST-1 dimer. This variant contains seven 3-bromotyrosines (purple sticks) and eight tyrosines (green sticks) per monomer. (c) Part of the crystal structure depicting domain I. The 3-bromotyrosines (Hal32, Hal56, and Hal73) are represented by the purple sticks, with the van der Waals radii of the bromines indicated by the purple spheres. Tyr56 and Asp59 are represented by the green sticks, with the dotted lines indicating interactions. The residues in contact with the bromines are represented by the sticks with the carbon, nitrogen, and oxygen atoms colored white, blue, and red, respectively. The α-4 helix (grey) belongs to the other monomer. (d) Part of the crystal structure around Hal57. The four residues in contact with the bromine moiety (a purple sphere) are represented by sticks. The 2FoFc map is shown around Hal57.

These halogenated GST variants (7iGST, 7bGST, and 7cGST) retained specific activities of 45, 47, and 60%, respectively, relative to that of wtGST. Heat resistance was observed for 7cGST, which retained 20% of its original activity after heating at 60°C for 10 min, whereas similar treatments inactivated wtGST, 7iGST, and 7bGST (Fig. 1a). The individual contributions of the 3-chlorotyrosines in 7cGST to the heat resistance were examined by changing them back to tyrosines separately. The removal of chlorine from two sites (positions 57 and 73) each almost abolished the heat resistance, indicating the stabilizing effects of chlorination at these positions. The effects of chlorination at four sites (positions −1, 22, 141, and 163) were nearly neutral. Interestingly, the chlorine removal from position 155 drastically increased the heat resistance of 7cGST, and the bromine removal from the same position elicited heat resistance from 7bGST. The bulky halogens incorporated at position 155 probably caused steric hindrance with neighboring residues.

The synergic effect of halogenation at different sites was revealed by comparing the GST variants chlorinated at one of positions 57, 73, and 141 and the variant chlorinated at the three positions simultaneously. This three-site variant (3cGST) was found to be more heat-stable than any of the single-site variants (Fig. 1a). Iodination and bromination at the same three sites (3iGST and 3bGST, respectively) also achieved significant heat resistance. Then, we examined the effects of additional modifications. The halogenation at position 32 significantly increased the heat resistance of 3bGST and 3cGST, whereas those at positions 27 and 56 each abolished the resistance. Positions 103, 110, 191, and 197 were moderately destabilizing or had neutral effects. The stabilizing/destabilizing nature of halogenation at each position was not affected by the halogenation at other positions. The opposite effects at positions 27 and 32 almost cancelled each other out in 3bGST(27b 32b) and 3cGST(27b 32b) (Fig. 1a), while additional halogenation at the neutral position 191 did not change the heat stabilities of the variants already halogenated at position 32 or positions 27 and 32 simultaneously [3bGST(32b 191b), 3cGST(32b 191b), 3bGST(27b 32b 191b), and 3cGST(27b 32b 191b) in Fig. 1a].

The distinction between stabilizing and destabilizing positions (Supplementary Table 1) and the synergy between different positions highlight the advantage of a method for incorporating synthetic amino acids at multiple selected sites. Finally, position 32 was halogenated and position 155 was made halogen-free in the initial variants 7bGST and 7cGST, to create 7bGST-1 and 7cGST-1, respectively. The heat resistance was drastically increased (0 to 71% for 7bGST-1, 11 to 79% for 7cGST-1) (Fig. 1a), and was accompanied by significant recoveries of the specific activity (47 to 67% for 7bGST-1 and 60 to 72% for 7cGST-1). The achieved stability was evaluated by measuring the unfolding curves against a chemical denaturant and determining the Gibbs free energy of unfolding (Supplementary Fig. 2; Supplementary Table 2). The Δ*G*(H_2_O) value determined for wtGST (27.7 kcal.mol) was almost equal to the reported value^11^. The Δ*G*(H_2_O) values for 7bGST-1 and 7cGST-1 (33.3 and 32.9 kcal/mol, respectively) were larger by 5.6 and 5.2 kcal/mol, respectively, than that of wtGST. Thus, the original stability of GST was increased 20% by the halogenation.

### Mechanism underlying the enhanced stability with bulky halogenation

To elucidate the mechanism underlying the structural stabilization, the crystal structures of 7bGST-1 and 7cGST-1 (PDB codes: 4WR4 and 4WR5, respectively) were determined at 1.6- and 1.9-Å resolutions, respectively (Fig. 1b; Supplementary Fig. 3; Supplementary Table 3). These structures superimposed well on the reported structure of wtGST^12^ (PDB code: 1UA5), with the root-mean-square deviations for the C_α_ atoms of 0.43 and 0.45 Å for 7bGST-1 and 7cGST-1, respectively. These small deviations indicate that the bulky halogens are incorporated with no significant changes in the main chain structures. The deviation was 0.089 Å between the variants. To evaluate the stabilizing effects of halogenation, we calculated the interaction energies for all of the residue pairs in the enzyme, using the *ab initio* fragment molecular orbital (FMO) method^13^. The interaction energy consists of two parts, due to the van der Waals and electrostatic interactions. The calculations were performed for the actual crystal structures, as well as their modified structures with the halogenated tyrosines virtually replaced by tyrosines. The interaction energies for the same residue pair were compared between these two types of structures, and the difference was interpreted as the effect of halogenation.

The major stabilizing positions 32, 57, and 73 are located in domain I, which consists of a four-stranded β-sheet and three α-helices (Fig. 1c). The side chains of the halogenated tyrosines at positions 32 and 57 (Hal32 and Hal57) protrude from each side of the β-sheet, and are buried in the protein interior. The halogen moiety of Hal32 fills the space between the side chains of Lys39 and Lys43 (Supplementary Fig. 4a, b), while that of Hal57 fills the space between the side chains of Pro2, Leu4, Leu20, and Tyr27 (Fig. 1d; Supplementary Fig. 5). The halogen moiety of Hal73 occupies the space at the dimer interface, between the side chains of Lys77 and Lys86, where Lys86 belongs to the other monomer (Supplementary Fig. 4c, d). The FMO calculation indicated that these halogen atoms form not only van der Waals contacts, but also electrostatic interactions, with the neighboring residues (Table 1; Supplementary Tables 4, 5). The halogens at the other positions (positions −1, 22, 141, and 163) also form similar interresidue interactions (Table 1; Supplementary Tables 4, 5), although stabilizing effects were barely detected in the heating experiments.

To obtain more insights into how the bulky halogens are accommodated in the protein interior, we evaluated the sizes of the spaces around the tyrosine *meta* positions. As described in the “Methods”, the space size was represented by the radius of a sphere just barely fits within the space (Supplementary Table 6). The halogen atoms are accommodated in spaces with sizes between 1.25 and 1.70 Å for wtGST, while the radii of the bromine and chlorine atoms are 1.85 and 1.75 Å, respectively. Significant increases in the space size (larger than 0.5 Å) were observed for the halogenated positions 32 and 141. Thus, the bulky halogens do not simply occupy vacant spaces that are large enough for the atoms, but fit into the spaces by causing local structural changes, probably involving side-chain movements, which cannot be identified from the crystal structures with the present resolutions.

The structural insights into domain I also revealed the network of canonical interactions connecting the three halogenated positions (Fig. 1c). Hal32 and Hal57 are connected via Tyr56, which forms a hydrogen bond with Hal32 between their hydroxyl groups and is also peptide-bonded to the neighboring Hal57. Hal57 is then connected with Hal73 via Asp59, which forms electrostatic interactions between its carboxyl group and the hydrogens of the hydroxyl groups of Hal57 and Hal73. These electrostatic interactions are strengthened by the halogenations at positions 57 and 73 (Table 1; Supplementary Tables 4, 5). This network of interactions probably augmented the individual effects of the halogenation at different positions, and thus strongly stabilizes domain I.

### Applicability of the revealed mechanism to various proteins

The structural study showed that the chlorination and bromination of the selected tyrosine residues achieved a more tightly packed protein interior, with the bulky atoms filling internal spaces and creating additional interresidue interactions. By contrast, fluorine is too small to exert such effects, and the strongly polarized C–F bond offers a different stabilization mechanism, as demonstrated in synthetic peptides assuming the leucine zipper configuration^14^,^15^. Although fluorinated amino acids can be chemically incorporated into peptides at desired sites, they have also been incorporated into proteins by the residue-specific method to totally replace one of the canonical amino acids, and with mixed results in terms of protein stabilization^16^,^17^,^18^,^19^.

**Figure 2:**
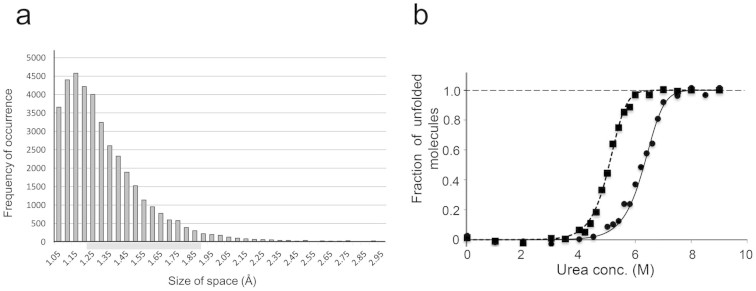
Applicability of bulky halogenation on tyrosine residues for protein stabilization. (a) Distribution of the sizes of the spaces near the 51,504 tyrosine *meta* positions in 2,168 proteins. There are 13,000 instances outside the indicated size range. The size range for the spaces potentially able to accommodate the bromine or chlorine atom is indicated by the grey bar. (b) Unfolding curves against a chemical denaturant for the wild-type azoreductase (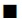, dotted line) and the three-site brominated variant (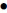, solid line). The fraction of unfolded molecules is plotted against the urea concentration.

In contrast, the site-specific incorporation of chlorine or bromine is considered to be an effective stabilization strategy for the following three reasons. First, there are many internal spaces that can be exploited by the halogens to exert stabilizing effects. Considering that the stabilizing halogens exploited internal spaces with sizes starting from 1.25 Å in GST, we assumed that those with sizes between 1.25 and 1.85 Å (the radius of bromine) are potentially available. A statistical analysis of 2,168 protein structures revealed that the spaces within this range account for 40% of the 51,504 tyrosine *meta* positions (Fig. 2a). Secondly, as exemplified in the present study, the hydroxyl group and the aromatic ring of tyrosine are frequently involved in forming the networks of interresidue interactions^20^, and could augment the individual effects of halogenation. Finally, tyrosine is abundantly present in industrially important proteins, such as oxidoreductases and the antigen-binding regions of antibodies.

We tested the utility of our strategy in an application to the industrially relevant azoreductase^21^,^22^. This *E. coli* enzyme is homodimeric^21^, and contains 7 tyrosines per 200-residue monomer, which is equal to the average tyrosine content (3.5%) in proteins. The seven tyrosines were first subjected to bromination individually. The variants brominated at positions 108, 156, and 179 each partially retained the specific activity after heating at 78°C for 10 min, whereas the wild-type enzyme was almost inactivated by this treatment (Supplementary Fig. 6). The simultaneous bromination at the three sites achieved a 13-fold longer half-life at the high temperature (Supplementary Fig. 7) and a 2.0-kcal/mol larger value for Δ*G*(H_2_O) (Fig. 2b; Supplementary Table 7), as compared with the non-halogenated enzyme. The finding of the three useful sites for stabilization was consistent with our calculation showing that roughly one-half of the tyrosine residues might be halogenated to increase stability. The successful and rapid stabilization of azoreductase demonstrated the applicability of our method, and this approach might be extended to include other types of halogenated amino acids.

The availability of various synthetic amino acids increases the utility of protein engineering based on the multiple selective-site incorporations of the amino acids. Specific translation machinery has been developed for each of >100 non-natural amino acids^1^,^2^,^3^. The space of protein sequences, expanded by combining the natural and non-natural diversity of amino acids, can now be explored by site-directed mutagenesis, structure-based design, and evolutionary methods^23^, to discover the novel structures and functionalities of proteins.

## Methods

### Protein preparation and heat-resistance assays

wGST and the GST variants containing 3-iodo-, 3-bromo-, and 3-chlorotyrosine were synthesized in the *E. coli* BW25113-based RFzero-iy strain^6^. The *gst* gene from *Schistosoma japonicum* had an additional amino-acid sequence, MASMTGGQQMGRDPGANSGVTKNSY, in place of the N-terminal Met. The tyrosine at the end of this sequence was numbered as −1. The Ser on the C-terminal side of this tyrosine was numbered as 1, corresponding to the numbering in the first report on the crystal structure of *S. japonicum* GST^12^. The plasmid pTacGST-IYN3 was previously constructed^6^. In brief, the *gst* gene was cloned in pET21b (Novagen), and the T7 promoter in the plasmid was then changed to the *tac* promoter. Finally, the genes encoding the archaeal tyrosyl-tRNA synthetase variant specific for halogenated tyrosines and the cognate UAG-reading tRNA were inserted downstream of *lacI* in the plasmid. *E. coli* BW25113-based RFzero-iy was transformed with pTacGST-IYN3, to synthesize wtGST and its variants. 3-Iodotyrosine (Sigma-Aldrich), 3-bromotyrosine (Watanabe Chemical Industries, Ltd., Japan), and 3-chlorotyrosine (Sigma-Aldrich) were supplemented at a concentration of 0.1 g/l in Terrific Broth (Novagen). GST was purified using a GST SpinTrap column (GE Healthcare Life Sciences), and then dissolved in buffer A [50 mM Tris-HCl (pH 8.0), 10 mM glutathione] at a concentration giving an optical density (OD) of 0.4 at 280 nm. An equal volume of glycerol was mixed with the solution before heat treatment. ESI-TOF-MS analyses were commercially performed by Mass Spectrometry Service, Research Resources Center, RIKEN Brain Science Institute (Wako, Japan).

The gene encoding *E. coli* azoreductase (AzoR) was C-terminally tagged with a hexa-histidine sequence and cloned in pET21b to create the plasmid pAzoR. *E. coli* BL21(DE3)-based RFzero-iy^6^ was transformed with pAzoR, and then cultured in Terrific Broth containing the Kao and Michayluk vitamin solution (Sigma-Aldrich), to synthesize the wild-type AzoR and its brominated variants. 3-Bromotyrosine was supplemented at 0.1 g/l. AzoR variants were purified using a His SpinTrap column (GE Healthcare Life Sciences), and then dissolved in buffer B [50 mM Tris-Cl (pH 7.4), 100 mM NaCl, 250 mM imidazole] at a concentration corresponding to an OD_280_ of 0.3. An equal volume of glycerol was mixed with the solution before heat treatment.

Heating was performed using a Veriti thermal cycler (Life Technologies). The specific activity of GST was measured using a GST Detection Module kit (GE Healthcare Life Sciences), according to the manufacturer's instructions, and an ARVO X3 photometer (Perkin Elmer) was used for determining the OD at 340 nm. The specific activity of AzoR was measured by mixing an aliquot of the AzoR solution (5 μl) with buffer C (100 μl) [100 mM Tris-Cl (pH8.5), 60 mM NaCl, 2 mM NADH, 0.1 mM tetrazole WST-8 (Dojindo Laboratories, Japan)] and incubating the mixture at room temperature for 5 min. The reactions with GST or AzroR were terminated by adding 100 μl of 0.1 M acetic acid.

### Thermodynamic analysis

Circular dichroism (CD) spectra were measured on a Jasco J-805 spectropolarimeter (JASCO Corp., Tokyo, Japan). The scan speed was 100 nm/min at a bandwidth of 1 nm. An average of four runs was recorded. Urea-induced equilibrium unfolding curves were obtained by recording the CD signal at 23°C and 222 nm. Unfolding experiments were performed in 20 mM sodium phosphate buffer, pH 6.5, containing 0.1 M NaCl and 2 mM DTT for GST, and in 25 mM Tris-HCl buffer, pH 7.4, for AzoR. The final concentrations of GST and AzoR monomers were 5.0 and 4.74 μM, respectively. The urea concentration of the stock solution was determined from refraction index measurements^24^, using an Abbe refractometer NAR-3T (Atago, Tokyo, Japan). The protein was incubated in the buffer with urea (0–8 M) for 1 h. All CD measurements were performed after equilibrium was attained.

Denaturation curves were evaluated according to the linear extrapolation method for a dimeric protein^25^. The denaturation equilibrium of dimeric proteins is assumed to be 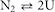. The equilibrium constant of this reaction, *K*_D_, was calculated at each point in the transition region of the denaturation curve by the equation: *K_D_* = [U]^2^/[N_2_] = 2*P_t_*[ *f_d_*^2^/(1 − *f_d_*)], where *P_t_* is the concentration of protein monomer, and *f_d_* is the fraction of unfolded protein. A linear dependence of the Gibbs free energy of unfolding (Δ*G_D_* = −RT · ln*K_D_*) on the denaturant concentration is assumed^26^: Δ*G_D_* = Δ*G_D_* (H_2_O) − *m* × [denaturant], where Δ*G_D_* (H_2_O) represents the difference in the Gibbs free energy between the unfolded and folded proteins in the absence of denaturant, and [denaturant] represents the denaturant concentration. The conformational stability parameters were determined by iterative fitting of denaturation curves to the above equations, using GraphPad Prism (GraphPad Software, La Jolla, CA) employing a Levenberg Marquard least-squares algorithm.

### Crystallization

The samples of the halogenated GST variants for crystallization were obtained by further purification, starting from the protein preparation used for heating experiments. The pooled fractions eluted with 10 mM reduced glutathione from affinity chromatography on an immobilized glutathione column (GE Healthcare Life Sciences) were applied to a Resource Q (1 mL) column, equilibrated with 50 mM Tris-HCl (pH 8.0) buffer containing 0.2 mM TCEP [*tris*(2-carboxyethyl)phosphine]. The variants were further purified using a 0–500 mM NaCl gradient with an AKTA 10 S system (GE Healthcare Life Sciences). Aliquots of each fraction were examined by SDS-PAGE. The pooled fractions were then concentrated with an AmiconUltra YM10 filter (Millipore) to 8 mg/ml, in 20 mM Tris-HCl (pH 8.0) buffer containing 50 mM NaCl, 1 mM reduced glutathione, and 1 mM TCEP. Crystallization was performed at 293 K by the sitting-drop vapor-diffusion method, in a drop of 0.3 μl protein solution (8.2 mg/ml) mixed with 0.3 μl reservoir solution, which was equilibrated against 100-μl reservoir solution on an Intelli plate (Art Robbins). The initial crystallization trial was performed using a commercial screening kit, NeXtal AmSO_4_ Suite (Qiagen). The optimized conditions were identified with a reservoir solution of 0.1 M tri-sodium citrate, 1.3 M ammonium sulfate, and 0.2 M lithium sulfate. Crystals were grown with a reservoir solution of 0.1 M MES (pH 7.0) buffer containing 2.0 M ammonium sulfate. Hexagonal-shaped crystals were obtained within a few days. An oil mixture (50:50 paraffin:paratone-N oil) was used as a cryoprotectant for the crystals.

### Data collection, structure determination and refinement

Synchrotron diffraction data were collected on the beamline BL26B2 at SPring-8, Harima, Japan. All data were processed using the XDS^27^ and XDSME^28^ software. Initial phasing was performed by molecular replacement with the PHASER software^29^, using the structure of the wild type GST (PDB ID: 1UA5) as a search model. A starting model was built into the electron density map, using the Coot software^30^, and was then refined using the PHENIX suite^31^. The stereochemical qualities of the final models were evaluated by the WHATIF^32^ and MolProbity^33^ programs.

### FMO calculations

We performed *ab initio* fragment molecular orbital (FMO) calculations to determine the interaction energy for each residue pair, as reported previously^34^. The calculations used the atomic coordinates from the crystal structures of 7bGST-1 and 7c-GST-1. The structures each comprised 218 residues (from positions −1 to 216), with seven halogenated tyrosine residues (positions 0, 22, 32, 57, 73, 141, and 163). The missing hydrogen atoms in 7bGST-1 and 7cGST-1 were added by using the molecular modeling software SYBYL-X. The orientations of the automatically added hydrogen atoms were optimized by energy minimization, using the Tripos force field. Three-dimensional data for non-halogenated GST [H-GST(b) and H-GST(c)] were constructed by replacing the halogen atoms with hydrogen atoms in the structures of 7bGST-1 and 7cGST-1, respectively. The orientations of the replaced hydrogen atoms were also optimized by energy minimization, using the Tripos force field. We performed FMO calculations for H-GST(b), H-GST(c), 7bGST-1, and 7cGST-1. In the FMO calculations, each GST structure was divided into one-residue fragments, with cut-off points at Cα of each residue. In addition, seven tyrosine residues (positions −1, 22, 32, 57, 73, 141, and 163) and a leucine residue (position 19) were divided into two fragments, with cut-off points at Cγ of each residue. All of these FMO calculations were performed using the PAICS program^35^ at the RI-MP2 level with the cc-pVDZ basis set. The HF and MP2 correlation energies correspond to electrostatic and van der Waals dispersion interactions, respectively. We analyzed the interaction energy for each residue pair by the pair interaction energy analysis based on the FMO calculations.

### Evaluation of the space size around the tyrosine *meta* positions

The atomic coordinates of the proteins to be analyzed were obtained from the Protein Data Bank; the selected molecules are all single-chain proteins with structures at resolutions between 2.0 and 2.5 Å and *R*_work_ values of <0.2, and the sequence identity between any pair from the selected proteins is <30%. Thus, the number of analyzed structures was 2,168, including 51,504 tyrosine *meta* positions. To determine the size of the vacant space around a given *meta* position, a sphere tangent to the center of the tyrosine Cε atom was assumed. The sphere radius was increased from 1.00 to 3.00 Å by 0.05 Å at each step, with the sphere center moving outward along the axis of the Cε-H bond. For each step, the possible formation of a van der Waals contact between the sphere and any atom of the neighboring residues was assessed. When there was a contact, the radius was recorded as the space size at the *meta* position. By iterating this procedure, a distribution of the space sizes near the *meta* positions was obtained. This process was also applied to calculate the sizes of the internal spaces near the tyrosine residues in GST.

## Author Contributions

K.O., A.Y. and T.M. synthesized halogenated protein variants and analyzed thermostability. H.K., N.H. and M.H. obtained thermodynamic parameters. S.K. and K.Y. performed FMO calculations. K.M. performed the statistical analysis. Y.T., T.I., R.A., M.K., C.T., M.S. and S.Y. performed the X-ray crystallography. K.S. conceived the study and wrote the manuscript.

## Additional Information

**How to cite this article**:Ohtake, K. *et al*. Protein stabilization utilizing a redefined codon. *Sci. Rep.* **5**, 9762; doi: 10.1038/srep09762(2015).

## Supplementary Material

Supplementary Information Supplementary information

## Figures and Tables

**Table 1:** Interresidue interactions enhanced by halogenation in 7bGST-1 and 7cGST-1.

Halogenated tyrosine	Interacting residues (Δ*E*_b_/Δ*E*_c_)*					
Hal−1	Pro2 (−0.1/−0.2)*	Ile3 (−1.7/−1.1)	Ile58 (−0.9/−0.6)			
Hal22	Leu19 (−0.8/−0.5)	Met80 (−0.8/−0.6)	His146 (−1.2/−0.9)	Val147 (−0.8/−0.8)	Phe152 (−0.2/−0.1)	
Hal32	Lys39 (−3.4/−2.6)	Trp40 (−0.6/−0.7)	Lys43 (−3.4/−2.2)			
Hal57	Pro2 (−0.7/−0.8)	Leu4 (−2.9/−1.9)	Leu20 (−0.4/−0.4)	Tyr27 (−0.7/−0.6)	Asp59 (−2.4/−1.3)	Ile74 (−0.3/−0.1)
Hal73	Asp59 (−3.7/−2.4)	Lys77 (−2.3/−0.1)	Lys86** (−3.9/−2.4)			
Hal141	Val147 (−0.6/−0.3)	Arg181 (−2.6/−0.9)	Ile182 (−0.9/−0.6)	Ile185 (−0.5/−0.5)		
Hal163	Trp200 (−0.5/−0.4)	Pro215 (−1.2/−1.0)	Pro216 (−0.7/−0.1)			

*The left- and right-side figures in the parentheses [Δ*E*_b_ and Δ*E_c_* (kcal/mol), respectively] indicate the stabilizing effects of bromination and chlorination, respectively, on the interactions between the residues shown above the parentheses and the halogenated tyrosines indicated on the far left. The separate contributions from van der Waals and electrostatic interactions are listed in Supplementary Tables 4 and 5.

**Lys86 is a residue from the other monomer.
