# Supplementary material for: Protein stabilization utilizing a redefined codon
Source: Sci Rep. 2015 May 18;5:9762. doi: 10.1038/srep09762 (PMC4434908; doi:10.1038/srep09762)
Supplement: Supplementary Information [file srep09762-s1.pdf]

**SUPPLEMENTARY INFORMATION**

# Protein stabilization utilizing a redefined codon

Kazumasa Ohtake, Atsushi Yamaguchi, Takahito Mukai, Hiroki Kashimura, Nobutaka Hirano, Mitsuru Haruki, Sosuke Kohashi, Kenji Yamagishi, Kazutaka Murayama, Yuri Tomabechi, Takashi Itagaki, Ryogo Akasaka, Masahito Kawazoe, Chie Takemoto, Mikako Shirouzu, Shigeyuki Yokoyama* & Kensaku Sakamoto*

*Authors for correspondence. E-mail: kensaku.sakamoto@riken.jp (K. S.), yokoyama@riken.jp (S. Y.); Phone: +81-45-503-9459 (K. S.), +81-45-503-9196 (S. Y.).

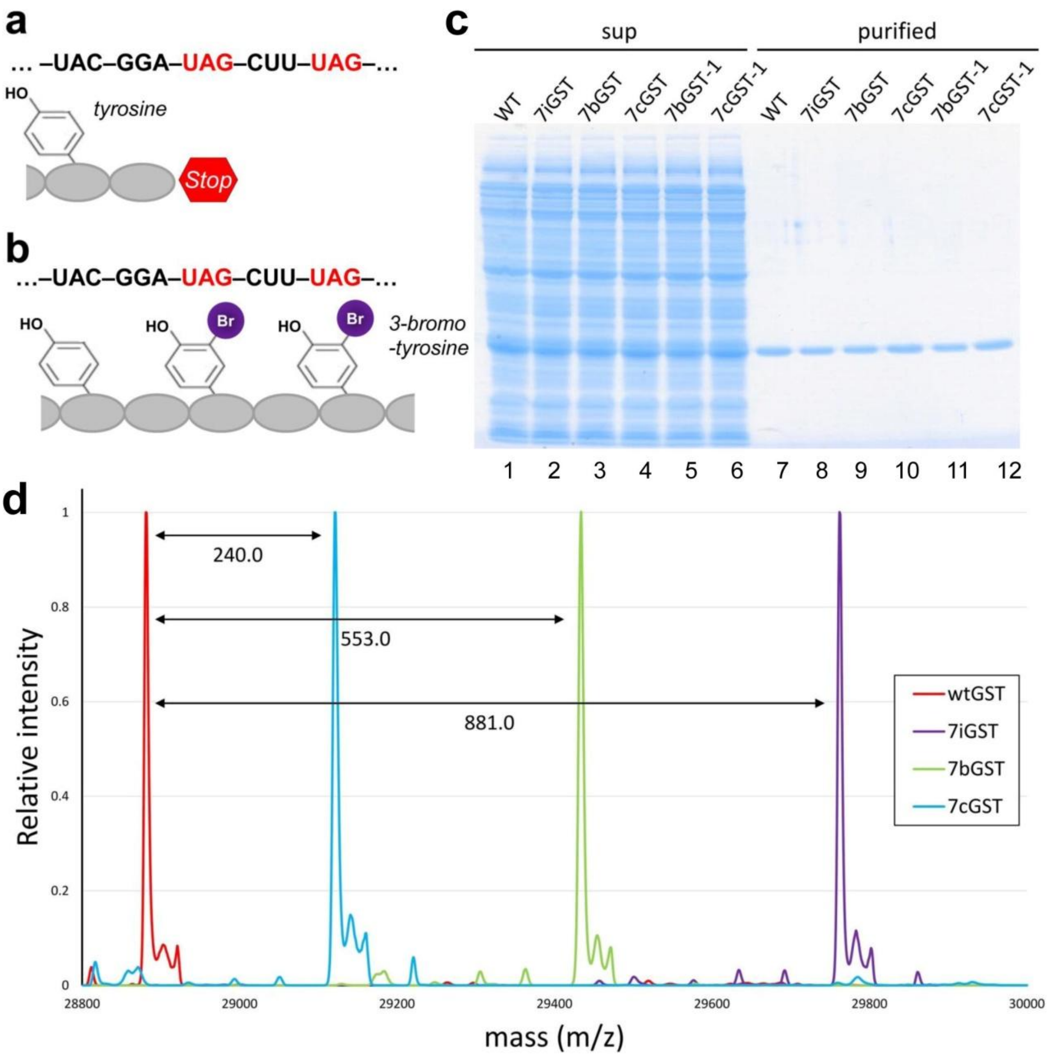


**Supplementary Figure 1 | UAG redefinition allows the incorporation of synthetic amino acids into proteins at multiple specific sites in the *E. coli* RFzero strain.** (**a**) Protein synthesis is terminated in response to the UAG stop codon in normal *E. coli* cells. (**b**) UAG is redefined as a sense codon specifying a bulky halogenated tyrosine in *E. coli* RFzero cells, and supports its efficient incorporation into proteins. Tyrosines are incorporated in response to UAU or UAC. (**c**) Biosynthesis of wtGST (WT) and its halogenated variants in RFzero-iy cells. The soluble protein fractions from the cells expressing the indicated GST molecules were analyzed in lanes 1—6. The purified products were analyzed in lanes 7—12. The proteins were detected by staining with Coomassie Brilliant Blue. (**d**) ESI-MS analyses of the purified wtGST and the indicated halogenated GST variants. The weight differences between wtGST and each of the variants correspond to the masses of seven atoms of the corresponding halogen, each minus that of seven hydrogens.

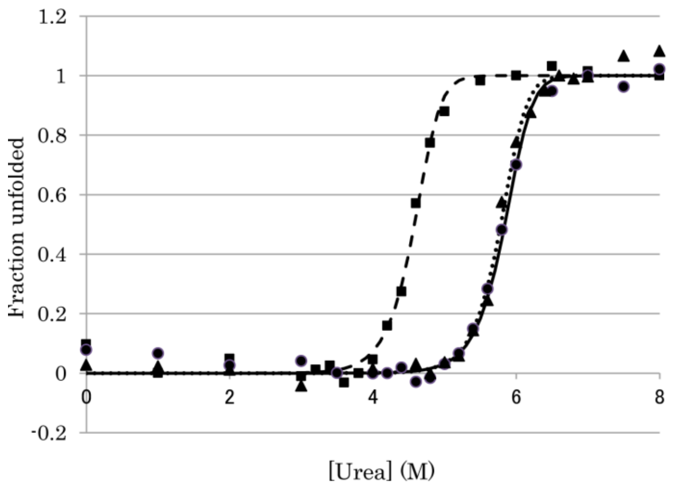


**Supplementary Figure 2 | Unfolding curves against a chemical denaturant (urea) for wtGST (■, dashed line), 7bGST-1 (●, solid line) and 7cGST-1(▲, dotted line).** The fraction of unfolded molecules is plotted against the urea concentration.

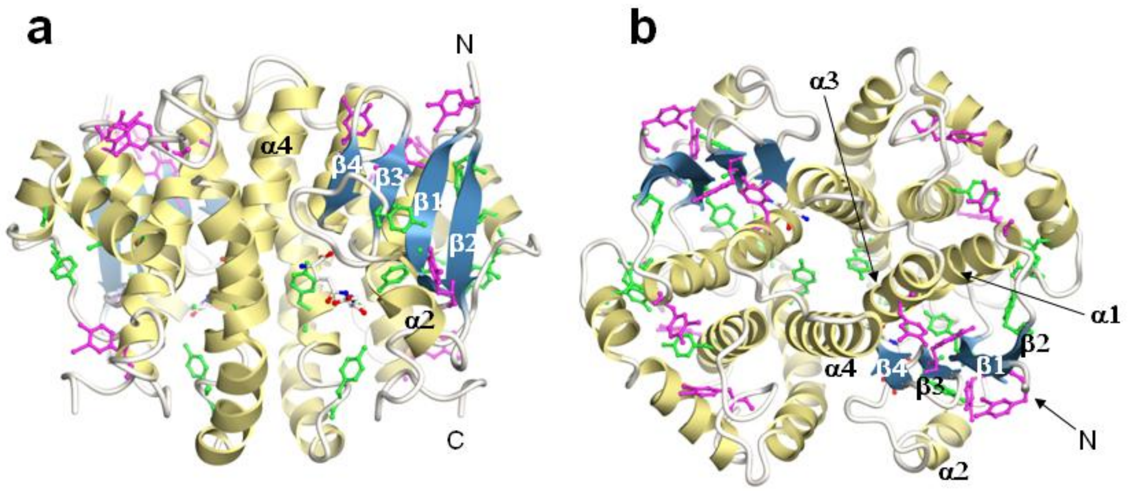


**Supplementary Figure 3 | Crystal structure of the 7bGST-1 dimer.** (**a**) Side view. (**b**) Top view. The side chains of the incorporated 3-bromotyrosines are represented by the purple sticks, while those of the tyrosine residues are represented by green sticks. The bound glutathione molecules are also represented by sticks, with the carbon, nitrogen, oxygen, and sulfur atoms colored white, blue, red, and green, respectively. Panel **a** is the same as Figure 1b.

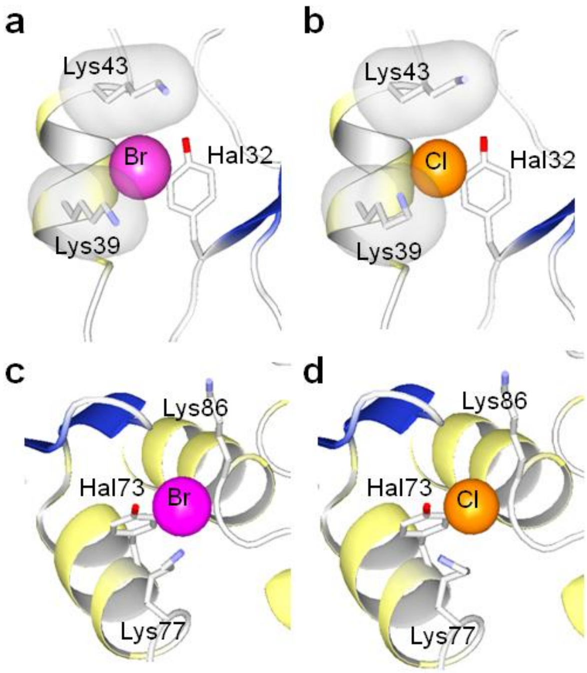


**Supplementary Figure 4 | Parts of the crystal structures depicting the 3-bromotyrosines (a, c) and 3-chlorotyrosines (b, d) at positions 32 (a, b) and 73 (c, d), together with the neighboring residues in contact with the halogens.** The side chains of these residues are represented by sticks with the carbon, nitrogen, and oxygen atoms colored white, blue, and red, respectively. The van der Waals radii of bromine and chlorine are depicted by purple and orange spheres, respectively. The surfaces for the side chains of Lys39 and Lys43 are indicated in white.

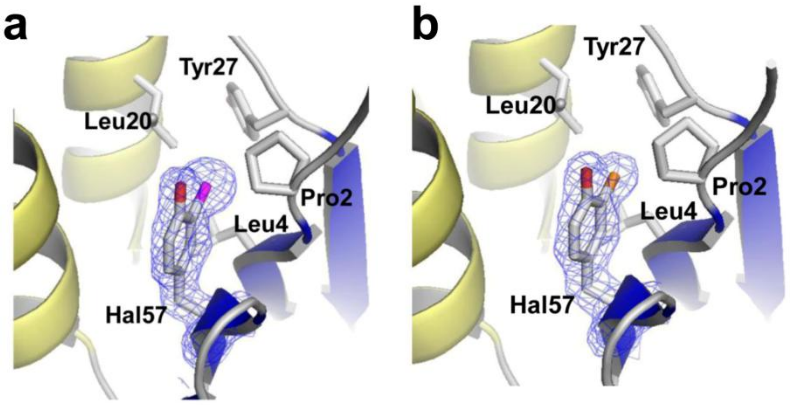


**Supplementary Figure 5 | Parts of the crystal structure depicting the 3-bromotyrosine (a) and 3-chlorotyrosine (b) at position 57, together the neighboring residues in contact with the halogens.** The side chains of these residues are represented by sticks, with the bromine and chlorine atoms colored purple and orange, respectively. The 2FoFc map is shown around Hal57. Panel **a** is the same as Fig. 1c.

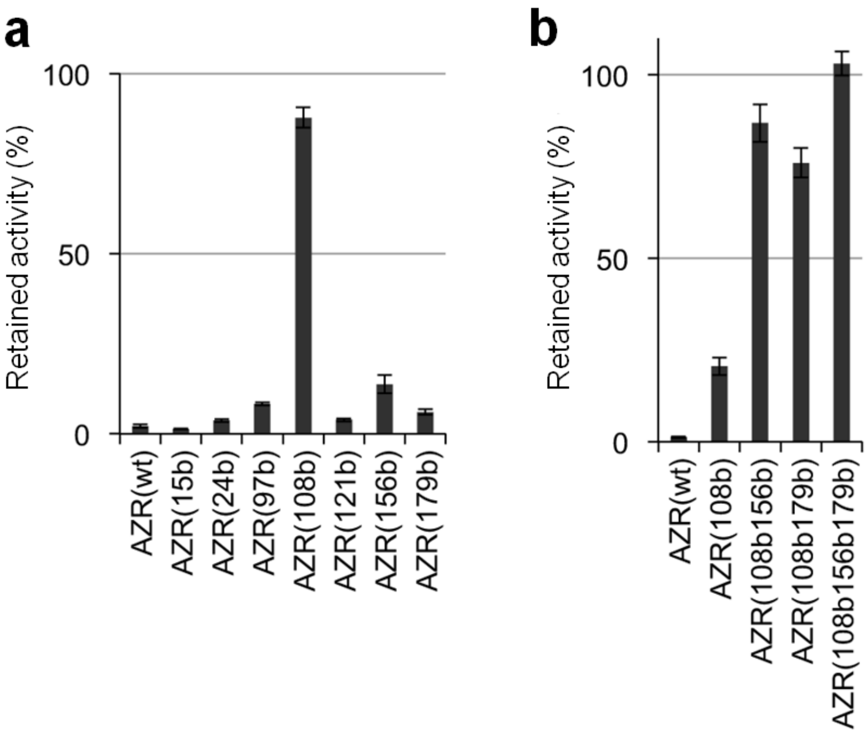


**Supplementary Figure 6 | Increasing the heat resistance of azoreductase (AZR) by incorporating 3-bromotyrosines.** The activities retained after heating at 78°C (**a**) and 80°C (**b**) for 10 min are shown for the wild-type enzyme (wt) and the brominated variants. The engineered sites are indicated in parentheses. The bromination at position 97 caused a drastic loss of specific activity, and the absolute level of the retained activity of AZR(97b) was correspondingly low. Error bars indicate standard deviations from three independent measurements for each variant.

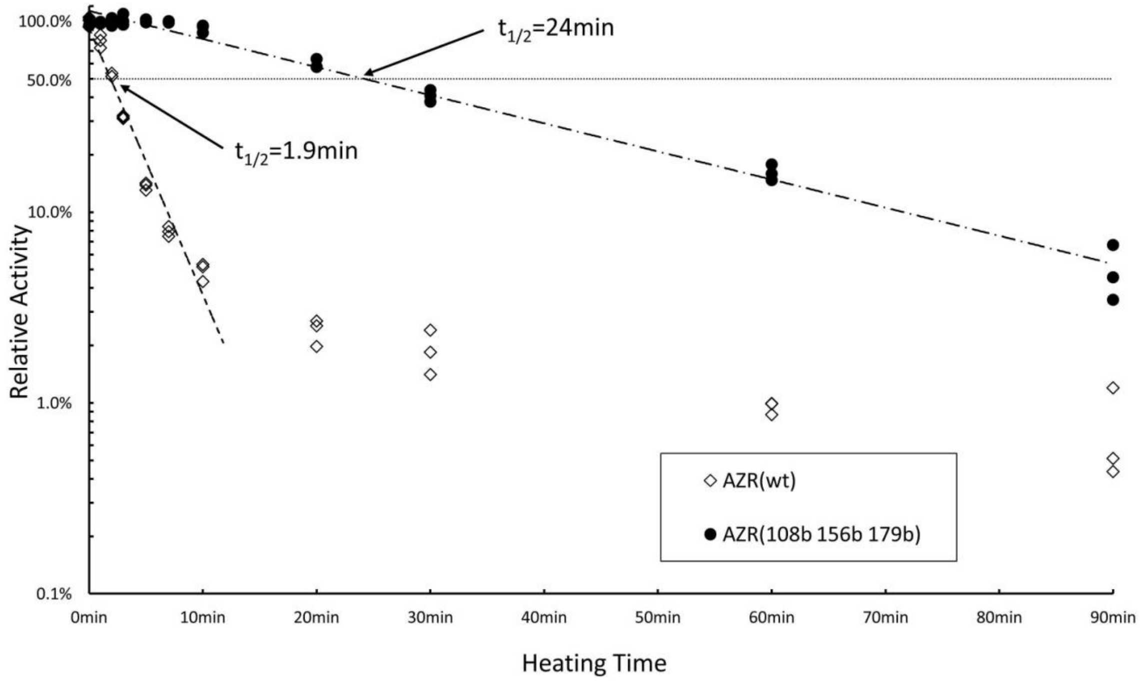


**Supplementary Figure 7 | Measurements of half-lives of the wild-type azoreductase from *E. coli* and its variant brominated at positions 108, 156, and 179.** Measurements were performed three times for each protein at 78°C. First-order fitting for the activity decaying curves gave 1.9 and 24 min as the half-lives of the wild-type and variant molecules, respectively.

**Supplementary Table 1 | Individual effects of halogenation at the 15 tyrosine positions of GST.**

| Position* | Location | Effect |
|---|---|---|
| −1†, ¶ | Surface | Neutral |
| 6 | Catalytic site | (Catalytically important, not engineered) |
| 22†, ¶ | Surface | Moderate |
| 27 | Buried | Destabilizing |
| 32¶ | Buried | Stabilizing |
| 56 | Buried | Destabilizing |
| 57†, ¶ | Buried | Stabilizing |
| 73†, ¶ | Dimer interface | Stabilizing |
| 103 | Buried | Neutral |
| 110 | Dimer interface | Neutral |
| 141†, ¶ | Surface | Moderate |
| 155† | Buried | Destabilizing |
| 163†, ¶ | Surface | Neutral |
| 191 | Buried | Neutral |
| 197 | Buried | Destabilizing |

*The numbering of the positions corresponds to that used in reference 10, except for position −1, which is included in the N-terminal tag.

†The initial variants (7iGST, 7bGST and 7cGST) were halogenated at these positions.

¶The final variants (7bGST-1 and 7cGST-1) were halogenated at these positions.

**Supplementary Table 2 | Thermodynamic parameters for wtGST, 7bGST-1, and 7cGST-1**

| | $\Delta G_D(H_2O)$ (kcal/mol) | $m$ (kcal/mol per M urea) |
|---|---|---|
| wtGST | 27.7 ± 0.2 | 4.55 ± 0.06 |
| 7bGST-1 | 33.3 ± 0.8 | 4.55 ± 0.16 |
| 7cGST-1 | 32.9 ± 0.2 | 4.43 ± 0.08 |

$*\Delta G_D = \Delta G_D (H_2O) - m \times [\text{denaturant}]$

**Supplementary Table 3 | Data collection and refinement statistics**

| | 7bGST-1 (PDB ID: 4WR4) | 7cGST-1 (PDB ID: 4WR5) |
|---|---|---|
| Wavelength (Å) | 1.000 | 1.000 |
| Resolution range (Å) | 44.51−1.601 (1.658−1.601) | 44.41−1.925 (1.994−1.925) |
| Space group | $P6_522$ | $P6_522$ |
| Unit-cell dimensions (Å, °) | *a*=*b*=63.85 *c*=225.02 | *a*=*b*=63.7 *c*=224.61 |
| No. of observations | 504183 (36290) | 446737 (44143) |
| No. of unique reflections | 36507 (3544) | 21466 (2094) |
| Multiplicity | 13.8 (10.2) | 20.8 (21.1) |
| Completeness (%) | 98.9 (99.1) | 100 (99.7) |
| $\langle I \rangle / \sigma(I)$ | 17.2 (1.11) | 22.7 (4.42) |
| Wilson B-factor | 21.1 | 21.9 |
| $R_{merge}$[a] (%) | 10.9 (213) | 11.7 (75.1) |
| $R_{meas}$ (%) | 11.4 (225) | 12.0 (76.9) |
| CC1/2 | 0.999 (0.627) | 0.999 (0.963) |
| CC* | 1 (0.878) | 1 (0.990) |
| $R_{work}$[b] (%) / $R_{free}$[c] (%) | 19.1/22.2 | 18.7/23.3 |
| Number of non-hydrogen atoms | 1968 | 1934 |
| No. of protein atoms | 1802 | 1802 |
| No. of GSH atoms | 20 | 20 |
| No. of $SO_4$ atoms | 10 | 10 |
| No. of water molecules | 136 | 102 |
| Protein residues | 218 | 218 |
| RMSD bonds (Å) | 0.010 | 0.009 |
| RMSD (angles) | 1.38 | 1.16 |
| Ramachandran favored (%) | 98 | 98 |
| Clash score | 1.12 | 0.56 |
| Average B-factor(Å$^2$) | 26.90 | 22.70 |
| Protein atoms | 26.50 | 22.50 |
| Non-protein atoms | 38.60 | 36.30 |
| Water molecules | 28.70 | 23.20 |

Values in parentheses are for the highest resolution shell.

[a] $R_{merge} = \sum_{hkl} \sum_i |I_i(hkl) - \langle I(hkl) \rangle| / \sum_{hkl} \sum_i I_i(hkl)$, where $I_i(hkl)$ is the intensity for the *i*th measurement of an equivalent reflection with indices *hkl*.

[b] $R_{work} = \sum_{hkl} ||F_{obs}| - |F_{calc}|| / \sum_{hkl} |F_{obs}|$; $F_{obs}$ and $F_{calc}$ are observed and calculated structure factor amplitudes, respectively.

[c] $R_{free}$ was calculated with randomly selected reflections (5%).

**Supplementary Table 4 | Interaction energies for the residue pairs involving the brominated tyrosines in 7bGST-1.** Interaction energies (kcal/mol) calculated using the structure of 7bGST-1 and its modified structure (H-GST), in which all of the halogens are virtually removed, are indicated, along with the differences between the interaction energies. The listed residues paired with 3-bromotyrosines are located within a 5.0-Å distance from the center of the bromine atoms.

| | | Interaction Energy | | | | Difference[a] | |
|---|---|---|---|---|---|---|---|
| | | H-GST | | 7bGST-1 | | | |
| Residue pair | | vdW[b] | Elec[c] | vdW | Elec | vdW | Elec (kcal/mol) |
| BrTyr−1 | Pro2 | −1.7 | −2.1 | −2.9 | −1.0 | −1.2 | +1.1 |
| BrTyr−1 | Ile3 | −0.2 | −0.4 | −1.1 | −1.2 | −0.9 | −0.8 |
| BrTyr−1 | Ile58 | −0.2 | +0.0 | −1.2 | +0.1 | −1.0 | +0.1 |
| BrTyr−1 | Asp59 | −0.3 | +2.2 | −2.2 | +6.1 | −1.9 | +3.9 |
| BrTyr−1 | Gly60 | −2.6 | +3.1 | −3.1 | +4.4 | −0.5 | +1.3 |
| BrTyr22 | Leu19 | −1.1 | −3.1 | −1.2 | −3.8 | −0.1 | −0.7 |
| BrTyr22 | Leu23 | −1.3 | +0.5 | −2.1 | +1.4 | −0.8 | +0.9 |
| BrTyr22 | Met80 | −0.4 | −0.2 | −2.1 | +0.7 | −1.7 | +0.9 |
| BrTyr22 | His146 | +0.0 | −1.8 | +0.0 | −3.0 | +0.0 | −1.2 |
| BrTyr22 | Val147 | −4.0 | −1.3 | −4.9 | −1.2 | −0.9 | +0.1 |
| BrTyr22 | Phe152 | −3.6 | +2.4 | −3.8 | +2.4 | −0.2 | +0.0 |
| BrTyr32 | Lys39 | −0.7 | −0.4 | −3.0 | −1.5 | −2.3 | −1.1 |
| BrTyr32 | Trp40 | −5.2 | +1.3 | −7.6 | +3.1 | −2.4 | +1.8 |
| BrTyr32 | Lys43 | −2.4 | −11.1 | −4.1 | −12.8 | −1.7 | −1.7 |
| BrTyr32 | Tyr56 | −4.0 | −4.3 | −4.1 | −3.8 | −0.1 | +0.5 |
| BrTyr57 | Pro2 | −3.2 | −0.4 | −4.2 | −0.1 | −1.0 | +0.3 |
| BrTyr57 | Ile3 | −0.4 | −0.7 | −0.9 | +0.4 | −0.5 | +1.1 |
| BrTyr57 | Leu4 | −3.2 | 1.3 | −4.3 | −0.5 | −1.1 | −1.8 |
| BrTyr57 | Leu20 | −0.5 | −0.2 | −2.3 | +1.2 | −1.8 | +1.4 |
| BrTyr57 | Tyr27 | −0.4 | 0.8 | −2.0 | +1.7 | −1.6 | +0.9 |
| BrTyr57 | Asp59 | −3.8 | −6.5 | −4.0 | −8.7 | −0.2 | −2.2 |
| BrTyr57 | Ile74 | −1.8 | −1.3 | −2.2 | −1.2 | −0.4 | +0.1 |
| BrTyr73 | Asp59 | −4.3 | −9.8 | −4.5 | −13.3 | −0.2 | −3.5 |
| BrTyr73 | Asp61 | −0.5 | +2.2 | −0.7 | +4.1 | −0.2 | +1.9 |
| BrTyr73 | Val62 | −4.4 | +0.6 | −5.7 | +2.7 | −1.3 | +2.1 |
| BrTyr73 | Lys77 | −4.2 | −4.3 | −5.3 | −5.5 | −1.1 | −1.2 |
| BrTyr73 | Pro85[d] | −0.7 | −0.3 | −2.4 | +1.8 | −1.7 | +2.1 |
| BrTyr73 | Lys86[d] | −1.0 | −4.6 | −4.7 | −4.8 | −3.7 | −0.2 |
| BrTyr141 | Val147 | −5.5 | +0.6 | −5.7 | +0.2 | −0.2 | −0.4 |
| BrTyr141 | Arg181 | −1.1 | −4.0 | −3.2 | −4.5 | −2.1 | −0.5 |
| BrTyr141 | Ile182 | −0.6 | +0.7 | −2.5 | +1.7 | −1.9 | +1.0 |
| BrTyr141 | Ile185 | −0.4 | +0.1 | −2.1 | +1.3 | −1.7 | +1.2 |
| BrTyr163 | Trp200 | −3.4 | +0.6 | −3.9 | +0.6 | −0.5 | +0.0 |
| BrTyr163 | Pro215 | −0.4 | −0.6 | −2.0 | −0.2 | −1.6 | +0.4 |
| BrTyr163 | Pro216 | −0.1 | −0.5 | −0.2 | −1.1 | −0.1 | −0.6 |

[a]Difference obtained for the residue pair indicated on the far left by subtracting the interaction energy for H-GST from that for 7bGST-1.

[b]Portion of the interaction energy due to the van der Waals interaction.

[c]Portion of the interaction energy due to the electrostatic interaction.

[d]Residue belonging to the other monomer.

**Supplementary Table 5 | Interaction energies for the residue pairs involving the chlorinated tyrosines in 7cGST-1.** Interaction energies (kcal/mol) calculated using the structure of 7cGST-1 and its modified structure (H-GST), in which all of the halogens are virtually removed, are indicated, along with the differences between the interaction energies. The listed residues paired with 3-chlorotyrosines are located within a 5.0-Å distance from the center of the chlorine atoms.

| | | Interaction Energy | | | | Differential energy[a] | |
|---|---|---|---|---|---|---|---|
| | | H-GST | | 7cGST-1 | | | |
| Residue pair | | vdW[b] | Elec[c] | vdW | Elec | vdW | Elec (kcal/mol) |
| ClTyr−1 | Pro2 | −1.7 | −2.1 | −2.4 | −1.6 | −0.7 | +0.5 |
| ClTyr−1 | Ile3 | −0.3 | −0.4 | −0.7 | −1.1 | −0.4 | −0.7 |
| ClTyr−1 | Ile58 | −0.4 | +0.0 | −1.0 | +0.0 | −0.6 | +0.0 |
| ClTyr−1 | Asp59 | −0.6 | +2.3 | −2.2 | +5.4 | −1.6 | +3.1 |
| ClTyr−1 | Gly60 | −3.5 | +5.0 | −4.0 | +6.2 | −0.5 | +1.2 |
| ClTyr22 | Leu19 | −1.1 | −3.3 | −1.2 | −3.7 | −0.1 | −0.4 |
| ClTyr22 | Leu23 | −1.7 | +0.7 | −2.4 | +1.5 | −0.7 | +0.8 |
| ClTyr22 | Met80 | −0.6 | −0.3 | −1.8 | +0.3 | −1.2 | +0.6 |
| ClTyr22 | His146 | +0.0 | −2.3 | +0.0 | −3.2 | +0.0 | −0.9 |
| ClTyr22 | Val147 | −4.0 | −1.2 | −4.6 | −1.4 | −0.6 | −0.2 |
| ClTyr22 | Phe152 | −3.4 | +1.9 | −3.5 | +1.9 | −0.1 | +0.0 |
| ClTyr32 | Lys39 | −1.1 | +1.0 | −2.6 | −0.1 | −1.5 | −1.1 |
| ClTyr32 | Trp40 | −5.2 | +0.9 | −7.0 | +2.0 | −1.8 | +1.1 |
| ClTyr32 | Lys43 | −2.6 | −6.0 | −3.9 | −6.9 | −1.3 | −0.9 |
| ClTyr32 | Tyr56 | −3.9 | −4.7 | −3.9 | −4.4 | +0.0 | +0.3 |
| ClTyr57 | Pro2 | −3.0 | −0.4 | −3.7 | −0.5 | −0.7 | −0.1 |
| ClTyr57 | Ile3 | −0.4 | −1.5 | −0.7 | −0.8 | −0.3 | +0.7 |
| ClTyr57 | Leu4 | −3.5 | +1.0 | −4.5 | +0.1 | −1.0 | −0.9 |
| ClTyr57 | Leu20 | −0.8 | −0.4 | −1.8 | +0.2 | −1.0 | +0.6 |
| ClTyr57 | Tyr27 | −0.5 | +1.0 | −1.7 | +1.6 | −1.2 | +0.6 |
| ClTyr57 | Asp59 | −2.8 | −2.0 | −2.9 | −3.2 | −0.1 | −1.2 |
| ClTyr57 | Ile74 | −2.3 | −0.9 | −2.5 | −0.8 | −0.2 | +0.1 |
| ClTyr73 | Asp59 | −4.5 | −11.6 | −4.7 | −13.8 | −0.2 | −2.2 |
| ClTyr73 | Asp61 | −0.7 | +2.5 | −0.8 | +3.5 | −0.1 | +1.0 |
| ClTyr73 | Val62 | −4.2 | +0.7 | −5.0 | +1.5 | −0.8 | +0.8 |
| ClTyr73 | Lys77 | −4.7 | −3.4 | −5.4 | −2.8 | −0.7 | +0.6 |
| ClTyr73 | Pro85[d] | −0.8 | −0.3 | −2.0 | +0.9 | −1.2 | +1.2 |
| ClTyr73 | Lys86[d] | −1.3 | −4.5 | −3.8 | −4.4 | −2.5 | +0.1 |
| ClTyr141 | Val147 | −5.4 | +0.3 | −5.6 | +0.2 | −0.2 | −0.1 |
| ClTyr141 | Arg181 | −1.4 | −3.9 | −3.1 | −3.1 | −1.7 | +0.8 |
| ClTyr141 | Ile182 | −0.8 | +0.6 | −2.1 | +1.3 | −1.3 | +0.7 |
| ClTyr141 | Ile185 | −0.7 | +0.0 | −1.7 | +0.5 | −1.0 | +0.5 |
| ClTyr163 | Trp200 | −4.0 | −0.4 | −4.3 | −0.5 | −0.3 | −0.1 |
| ClTyr163 | Pro215 | −0.5 | −0.6 | −1.5 | −0.6 | −1.0 | +0.0 |
| ClTyr163 | Pro216 | −0.2 | −0.2 | −0.3 | −0.2 | −0.1 | +0.0 |

[a]Difference for the residue pair indicated on the far left obtained by subtracting the interaction energy for H-GST from that for 7cGST-1.

[b]Portion of the interaction energy due to the van der Waals interaction.

[c]Portion of the interaction energy due to the electrostatic interaction.

[d]Residue belonging to the other monomer.

**Supplementary Table 6 | Sizes of the spaces around the tyrosine *meta* positions in wGST, 7bGST-1, and 7cGST-1.** The *meta* positions halogenated in 7bGST-1 and 7cGST-1 are marked with the asterisks. Position 73, located at the dimer interface, was excluded from the calculation.

| Residue | wGST (Å) | 7bGST-1(Å) | 7cBST-1(Å) |
|---|---|---|---|
| −1 | — | 3.00 | 3.00 |
| −1* | — | 1.90 | 1.70 |
| 6 | 1.20 | 1.20 | 1.15 |
| 6 | 1.05 | 1.10 | 1.10 |
| 22 | 1.45 | 1.50 | 1.50 |
| 22* | 1.70 | 1.80 | 1.75 |
| 27 | 1.05 | 1.15 | 1.00 |
| 27 | 1.05 | 1.05 | 1.05 |
| 32 | 1.20 | 1.20 | 1.30 |
| 32* | 1.35 | 2.00 | 1.90 |
| 56 | 1.35 | 1.30 | 1.35 |
| 56 | 1.05 | 1.15 | 1.10 |
| 57 | 1.30 | 1.10 | 1.25 |
| 57* | 1.45 | 1.95 | 1.80 |
| 103 | 1.00 | 1.20 | 1.20 |
| 103 | 1.00 | 1.20 | 1.20 |
| 110 | 1.05 | 1.50 | 1.35 |
| 110 | 1.50 | 1.45 | 1.40 |
| 141 | 1.35 | 1.10 | 1.15 |
| 141* | 1.25 | 1.95 | 1.80 |
| 155 | 1.05 | 1.05 | 1.10 |
| 155 | 1.30 | 1.50 | 1.35 |
| 163 | 1.25 | 1.10 | 1.15 |
| 163* | 1.70 | 2.00 | 1.95 |
| 191 | 1.15 | 1.15 | 1.10 |
| 191 | 1.10 | 1.50 | 1.50 |
| 197 | 1.15 | 1.30 | 1.20 |
| 197 | 1.25 | 1.15 | 1.20 |

**Supplementary Table 7 | Thermodynamic parameters for the wild-type AzoR and the variant brominated at positions 108, 156, and 179.**

| | $\Delta G_D(H_2O)$ (kcal/mol) | $m$ (kcal/mol per M urea)* |
|---|---|---|
| Wild-type | 20.6 ± 0.2 | 2.61 ± 0.04 |
| Variant | 22.6 ± 0.3 | 2.44 ± 0.05 |

$*\Delta G_D = \Delta G_D\ (H_2O) - m \times [\text{denaturant}]$

**Supplementary Table 8 | Oligonucleotides used in this study.**

For changing tyrosine codons to UAG in GST

| Position | Primer Name | Sequence (from 5' to 3') |
|---|---|---|
| 27 | GST_Y27Amb_fwd | aaaaaTAGgaagagcatttgtatgag |
| 27 | GST_Y27Amb_rev | gctcttcCTAtttttcttcaagctat |
| 32 | GST_Y32Amb_fwd | atttgTAGgagcgcgatgaaggtgat |
| 32 | GST_Y32Amb_rev | cgcgctcCTAcaaatgctcttcatat |
| 56 | GST_Y56Amb_fwd | ttcctTAGtagattgatggtgatgtt |
| 56 | GST_Y56Amb_rev | caatctaCTAaggaagattgggaaac |
| 103 | GST_Y103Amb_fwd | ttagaTAGggtgtttcgagaattgca |
| 103 | GST_Y103Amb_rev | aaacaccCTAtctaatatccaaaacc |
| 110 | GST_Y110Amb_fwd | ttgcaTAGagtaaagactttgaaact |
| 110 | GST_Y110Amb_rev | ctttactCTAtgcaattctcgaaaca |
| 191 | GST_Y191Amb_fwd | ataagTAGttgaaatccagcaagtat |
| 191 | GST_Y191Amb_rev | atttcaaCTActtatcaatttgtggg |
| 197 | GST_Y197Amb_fwd | gcaagTAGatagcatggcctttgcag |
| 197 | GST_Y197Amb_rev | atgctatCTActtgctggatttcaag |

For back replacements from UAG to tyrosine codons in GST

| Position | Primer Name | Sequence (from 5' to 3') |
|---|---|---|
| 22 | GST_Amb22Y_fwd | tggaaTATcttgaagaaaaatatgaa |
| 22 | GST_Amb22Y_rev | cttcaagATAttccaaaagaagtcga |
| 57 | GST_Amb57Y_fwd | cttatTATattgatggtgatgttaaa |
| 57 | GST_Amb57Y_rev | catcaatATAataaggaagattggga |
| 73 | GST_Amb73Y_fwd | tacgtTATatagctgacaagcacaac |
| 73 | GST_Amb73Y_rev | cagctatATAacgtatgatggccata |
| 141 | GST_Amb141Y_fwd | aaacaTATttaaatggtgatcatgta |
| 141 | GST_Amb141Y_rev | catttaaATAtgttttatgacataaa |
| 155 | GST_Amb155Y_fwd | tgttgTATgacgctcttgatgttgtt |
| 155 | GST_Amb155Y_rev | gagcgtcATAcaacatgaagtcagga |
| 163 | GST_Amb163Y_fwd | ttttaTACatggacccaatgtgcctg |
| 163 | GST_Amb163Y_rev | ggtccatGTAtaaaacaacatcaaga |

pAzoR plasmid construction

| Primer Name | Sequence (from 5' to 3') |
|---|---|
| His-pET21b-ifF | catcaccacc accatcacTA Agatccggct gctaacaaag cccga |

| | |
|---|---|
| pET21b-ifR | ATGTATATCT CCTTCTTAAA GTTAAACAAA |
| pET21b-EcAzoR-ifF | GAAGGAGATA TACATatgag caaggtatta gttcttaaat ccagc |
| pET21b-EcAzoR-ifR | atggtggtggtgatgTCCAGATCCtgcagaaacaatgctgtcgat |

The first two primers were used for amplifying the pET21b vector. The last two primers were used for amplifying the *azoR* gene together with the C-terminal tag

For changing tyrosine codons to UAG in AzoR

| Position | Primer Name | Sequence (from 5' to 3') |
|---|---|---|
| 15 | EcAzoR_Y15Amb_fwd | cagggTAGtctcagtctaatcagttg |
| 15 | EcAzoR_Y15Amb_rev | actgagaCTAccctgccaggatgctg |
| 24 | EcAzoR_Y24Amb_fwd | ccgatTAGtttgttgaacaatggcgc |
| 24 | EcAzoR_Y24Amb_rev | caacaaaCTAatcggacaactgatta |
| 97 | EcAzoR_Y97Amb_fwd | cgatgTAGaacttcaacatctcgact |
| 97 | EcAzoR_Y97Amb_rev | tgaagttCTAcatcggtgccgcaata |
| 108 | EcAzoR_Y108Amb_fwd | aaaatTAGtttgacctggttgcccgc |
| 108 | EcAzoR_Y108Amb_rev | ggtcaaaCTAatttttcaactgagtc |
| 121 | EcAzoR_Y121Amb_fwd | tccgcTAGaccgagaacggtccggaa |
| 121 | EcAzoR_Y121Amb_rev | tctcggtCTAgcggaaagtaacgcct |
| 156 | EcAzoR_Y156Amb_fwd | cgccgTAGctgtccacgttcctcggc |
| 156 | EcAzoR_Y156Amb_rev | tggacagCTAcggcgtcaccaggtcc |
| 179 | EcAzoR_Y179Amb_fwd | tcgcaTAGggtccggaaatggcagcg |
| 179 | EcAzoR_Y179Amb_rev | ccggaccCTAtgcgatcccttcggcg |
